# Automated Image Analysis Pipeline Development to Monitor Disease Progression in Muscular Dystrophy Using Cell Profiler

**DOI:** 10.26502/ami.936500115

**Published:** 2023-09-01

**Authors:** Alexandra Brown, Brooklyn Morris, John Karanja Kamau, Abdullah A. Alshudukhi, Abdulrahman Jama, Hongmei Ren

**Affiliations:** 1 Department of Biochemistry and Molecular Biology, Wright State University, Dayton, OH, USA; 2 Department of Medical Laboratories, College of Applied Medical Sciences, Qassim University, Buraydah, Saudi Arabia

**Keywords:** Evan’s Blue Dye, CellProfiler, Automated Histopathology Analysis, Muscle Regeneration, Muscular Dystrophy, Fibrosis

## Abstract

Muscular dystrophies are inherited disorders that are characterized by progressive muscle degeneration. These disorders are caused by mutations in the genes encoding structural elements within the muscle, which leads to increased vulnerability to mechanical stress and sarcolemma damage. Although myofibers have the capacity to regenerate, the newly formed myofibers still harbor genetic mutation, which induces continuous cycles of muscle fiber death and regeneration. This repeated cycling is accompanied by an inflammatory response which eventually provokes excessive fibrotic deposition. The histopathological changes in skeletal muscle tissue are central to the disease pathogenesis. Analysis of muscle histopathology is the gold standard for monitoring muscle health and disease progression. However, manual, or semi-manual quantification methods, are not only immensely tedious but can be subjective. Here, we present four image analysis pipelines built in CellProfiler which enable users without a background in computer vision or programming to quantitatively analyze biological images. These image analysis pipelines are designed to quantify skeletal muscle histopathological staining for membrane damage, the abundance and size distribution of regenerating muscle fibers, inflammation via quantification of CD68+ M1 macrophages, and collagen deposition. Additionally, we discuss methods to address common errors associated with the quantification of microscopy images. These automated tools can not only improve workflow efficiency but can provide a better understanding of the histopathological progression of muscular dystrophy.

## 1. Background

Muscular dystrophies consist of a group of inherited disorders that cause progressive muscle weakness and atrophy due to defects in the muscle structural support genes [1]. Duchenne muscular dystrophy (DMD) is the most common type of inherited muscular dystrophy, affecting approximately 1 in 3,500 to 1 in 5,000 male births worldwide [2, 3]. Patients with DMD typically become wheelchair-dependent around 10–12 years of age and most patients die in their early thirties due to cardiac and/or respiratory failure [4]. DMD is caused by a mutation in the dystrophin gene, which is located on the X-chromosome [5]. In muscle cells, the dystrophin protein connects the muscle contractile apparatus to the extracellular matrix [2]. In the absence of dystrophin, the sarcolemma becomes susceptible to mechanical stress during muscle contraction. In fact, these membrane disruptions can be visualized via the release of intracellular enzymes [6] and the uptake of large molecules and dyes, such as Evans Blue Dye [7].

In response to damage, skeletal muscles have the capacity to regenerate new muscle fibers [8]. The presence of regenerating muscle fibers can easily be detected by their re-expression of developmental isoforms of muscle proteins, such as embryonic myosin heavy chain (eMyHC) [9]. eMyHC can be detected 2–3 days following muscle injury, and its expression persists for about 2–3 weeks post-injury [9]. Once the regenerative process is complete, the size of the newly formed muscle fibers increases, and the nuclei migrate to the cell periphery [10]. The success of the regenerative process is dependent on a balance between pro-inflammatory and anti-inflammatory factors, which determine whether the damaged site will be replaced by new myofibers or by fibrotic tissue [11].

The chronic nature of DMD causes persistent inflammation which further promotes disease progression [12]. Necrotic areas within the muscle typically display an abundance of CD68+ M1 macrophages, which are the first macrophages to invade the injured muscle tissue [13, 14]. Sustained inflammation within the muscle induces excessive deposition of extracellular matrix components often in place of new myofibers [12]. Even when the new muscle fibers are successfully regenerated in the dystrophic muscle, these new fibers are prone to degeneration because they still harbor the dystrophin mutation; thus, triggering repeated cycles of muscle fiber degeneration, chronic inflammation, and significant collagen deposition [12]. With the advancement of the disease, patients present progressive replacement of muscle tissue with fibrous and adipose tissue [15]. Together these pathological changes lead to a profound loss of muscle function.

Assessing muscle fiber damage, regeneration, inflammation, and fibrosis is essential to monitor the progression of muscular dystrophies. However, quantitative analysis of these pathologies from histological and immuno-staining often involves a labor-intensive manual process. We introduce four image analysis pipelines which were created in CellProfiler to quantify these characteristics of skeletal muscle disease. CellProfiler enables large-scale image processing without the need for any prerequisite knowledge of programming languages. In addition, it is a free open-source software that is available for both Windows and MacOS.

## 2. Methods

### 2.1 Animals

The study is reported in accordance with ARRIVE guidelines (https://arriveguidelines.org). C57BL/10ScSnJ (B10, #000476) wildtype (WT) and C57BL/10ScSn-Dmdmdx/J (*mdx*, #001801) mice were purchased from Jackson Laboratories (Bar Harbor, ME, USA) and were used as WT control and DMD model for all experiments. As DMD occurs primarily in males, only male mice were used in the current study. These mice had free access to drinking water and regular chow, unless otherwise noted. All animal experiments were performed in accordance with the relevant guidelines and regulations approved by the Animal Care and Use Committee of Wright State University.

### 2.2 Tissue Collection and Sectioning

The gastrocnemius muscles were snap frozen using isopentane chilled with liquid nitrogen prior to embedding in Optimal Cutting Temperature (OCT) (Tissue-Tek, Sakura-Americas). The frozen tissues were stored at −80°C. Gastrocnemius muscles were sectioned at 10μM using a Thermo Fisher cryostat micron HM550 set at −24°C. Slides were then stored at −20°C until subsequent experimentation.

### 2.3 Immunofluorescence and Microscopy Image Acquisition

Muscle sections were air dried for 1h at room temperature. The tissue sections used to detect muscle regeneration were first blocked with 1XPBS containing mouse-on-mouse blocking reagent (#MKB-2213; Vectashield) for 1h at room temperature. Thereafter, tissue sections were blocked in 1XPBS containing 3% BSA (#BP9704; Thermo Fisher Scientific, Waltham, MA, USA) for 1h at room temperature. To detect muscle regeneration, tissue sections were incubated with antibodies against laminin 1α (ab11575; dilution 1:400; Abcam) and eMyHC (F1.652; dilution 3μg/μl; Developmental Studies Hybridoma Bank, Iowa City, IA, USA) for 45 minutes at 37°C. To detect muscle inflammation, tissues were first blocked in 1XPBS containing 3% BSA (#BP9704; Thermo Fisher Scientific, Waltham, MA, USA) for 1h at room temperature, then incubated with antibodies against CD68 (#97778; dilution 1:500; Cell Signaling Technology) and laminin 1α (ab11575; dilution 1:400; Abcam). All tissue sections were subsequently incubated with an Alexa Fluor 488- or Alexa Fluor 555-conjugated secondary antibody (#A-21411; Thermo Fisher Scientific) for 1h in the dark at room temperature. Nuclei of cells were detected by applying Vectashield antifade mounting medium with 4,6-diamidino-2-phenylindole (DAPI) (Vector Laboratories; H-1200-10). Images were obtained using an inverted microscope (IX70 Olympus) equipped with a DFC7000T camera (Leica Microsystems, Wetzlar, Germany). Indicated images were quantified using the CellProfiler software.

### 2.4 Evans Blue Dye (EBD) Assay

EBD dye injection was performed as we did previously [16, 17]. Briefly, mice were injected with EBD (10mg/ml stock in sterile saline, 0.1ml/10g body weight) I.P. and euthanized 24h later. The skeletal muscles were dissected and snap-frozen in isopentane cooled Optimal Cutting Temperature (OCT) embedding media (Tissue-Tek, Sakura-Americas). Frozen OCT blocks were cryo-sectioned at 10μM thickness and stained with laminin 1α (ab11575; dilution 1:400; Abcam) antibody before being analyzed by fluorescence microscopy.

### 2.5 Histology Staining to Assess Fibrosis

Fibrosis was assessed using Picrosirius red staining. Muscle tissue sections were washed twice in xylene for 5 minutes, twice in 100% ethanol, twice in 95% ethanol, and twice in 70% ethanol for 30 seconds each wash. Then tissue sections were washed in dH2O for 5 minutes. Picrosirius red dye (ab150681; Abcam) was applied on top of each tissue section and allowed to incubate at room temperature for 1h. Slides were then quickly dipped in dH2O 3 times. Afterward, slides were washed twice in 0.5% acetic acid for 30 seconds, then dehydrated three times in 100% ethanol for 15 seconds. Once the slides were dry, toluene solution (Fisher Chemical; SP15-500) was used as the mounting medium, and coverslips were applied. Images were obtained using an EVOS brightfield microscope.

### 2.6 Automated Image Analysis Using CellProfiler

CellProfiler is a free open-source software which relies on advanced statistical algorithms to quantitatively analyze biological images. CellProfiler provides a user-friendly platform for the design of image analysis pipelines which can easily be adapted for a variety of use cases. This software can be downloaded from the CellProfiler website (https://cellprofiler.org/releases) and will run on Windows or Mac operating systems. For the work presented here, version 4.2.1 was utilized, however, a newer version of the software is now available (4.2.4). After the software was downloaded, a sequential set of individual modules was pieced together to form the image analysis pipelines. Before testing out the software, it is recommended to review the CellProfiler manuals, which can be found at (https://cellprofiler.org/manuals).

### 2.7 Statistical analysis

Statistical analysis was performed with an unpaired t-test to determine significant changes between WT and *mdx* groups. All statistical analyses were performed using GraphPad Prism 9 (GraphPad Software, version 9.4.0, CA, USA). Data are provided as the mean ± SD number (n) of independent experiments. For all analyses, p < 0.05 was considered statistically significant. The four quantification pipelines for assessing muscle fiber damage, regeneration, inflammation, and fibrosis can be downloaded from the Supplementary Materials section.

## 3. Results

### 3.1 Quantification of Muscle Membrane Damage Using EBD Staining

**Figure 1:**
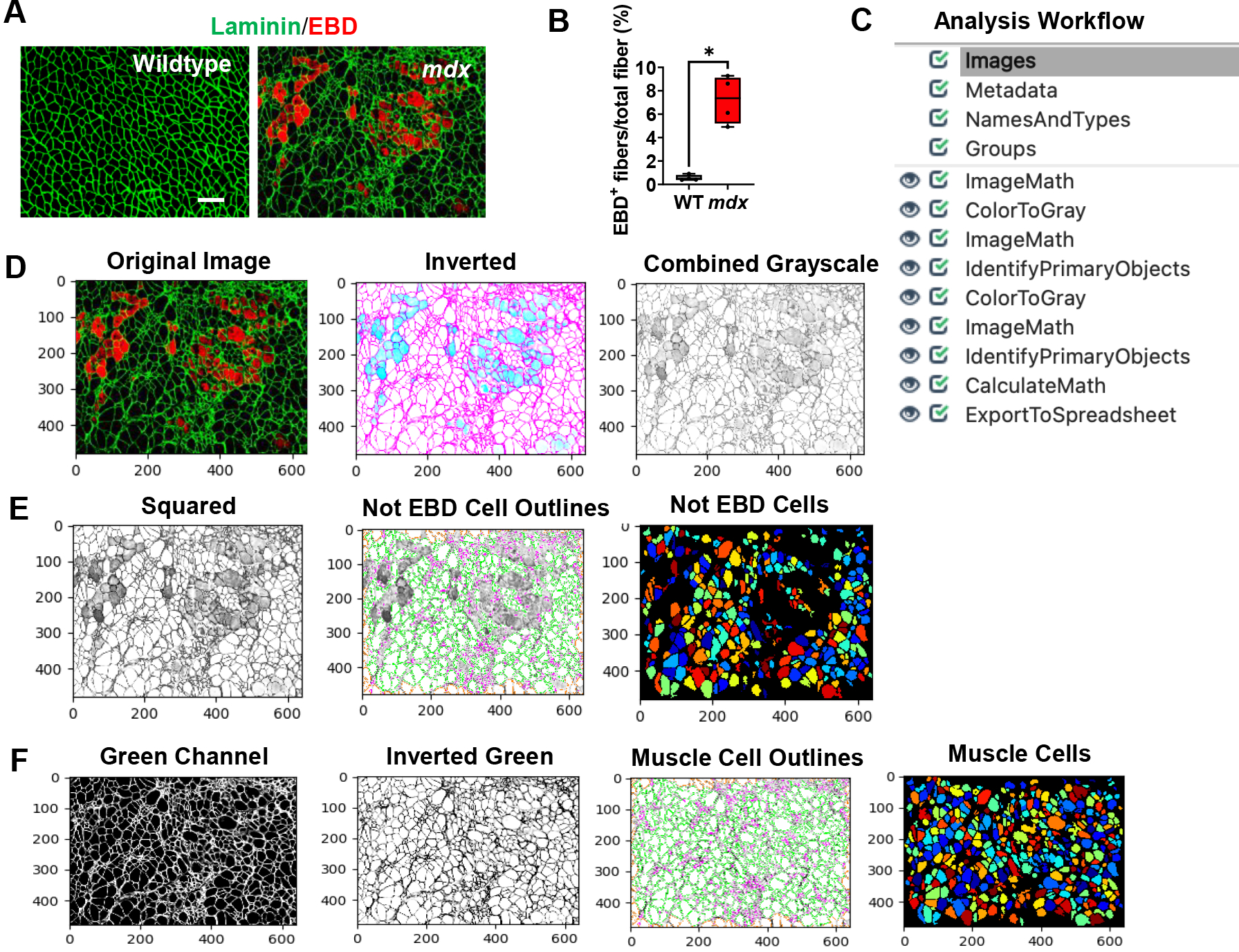
Processing and quantification of EBD+ myofibers by CellProfiler. **A)** Representative images of gastrocnemius muscle cross-sections from 4–6-month-old B10 and *mdx* mice immunolabeled with laminin α (green), and EBD (red). Scale bar = 100μm. **B)** Quantification analysis of EBD-positive muscle fiber expressed as the percentage of the total number of muscle fibers in WT and mdx mice (n = 5 mice/group). **p<0.01. **C)** Overview of the image analysis workflow. **D)** A sample *mdx* image was fed into the EBD quantification pipeline, which first inverts the original image. The inverted image is then converted into a grayscale image with the red and green channels combined. The pixels are then squared to mask the EBD+ cells. The “not” EBD cells are then identified and counted. **E)** The original image is retrieved and split into separate grayscale images representing each of the color channels (red, green, and blue). **F)** The green channel grayscale image is then inverted and used for subsequent identification of all muscle cells.

In muscular dystrophies, muscle membrane damage can be detected through staining with EBD, a membrane-permeable marker. After quantifying our sample EBD datasets, we found that nearly 7% of the myofibers in the *mdx* gastrocnemius muscle were EBD+, while the WT muscle didn’t exhibit any EBD+ fibers (Figure 1A & 1B, *p value* 0.0286). To perform this quantification, gastrocnemius muscles of WT and *mdx* mice were co-stained with EBD and laminin. All microscopy images were saved as TIFF files. It is recommended to save images in either TIFF or PNG formats, which do not lose information during format conversion, however, CellProfiler will accept a variety of image formats. An overview of the EBD quantification pipeline is shown in Figure 1C and can be downloaded from the Supplementary Materials section (Supplementary File S1). To begin quantification, the images to be quantified must first be loaded into the input panel. One advantage to using CellProfiler is that it will accept as many files or folders as needed.

Once the images are loaded as input, the user can then load an existing pipeline or create one from scratch. The image analysis pipeline that was created for this work performs general image processing functions such as image manipulation using arithmetic operations and object identification. One of the challenges of quantifying EBD+ myofibers is that membrane damage leads to poorly defined myofiber boarders. Instead of simply using the red channel to quantify the EBD+ myofibers, we first masked the EBD+ cells and identified the cells which didn’t exhibit any EBD staining. We then subtracted this quantity from the total number of cells in the image to give the number of EBD+ cells. An overview of the EBD image analysis workflow is described below:

1) As shown in Figure 1D, once an EBD immunofluorescence image has been loaded into the software, the pipeline will first invert the input image using the “ImageMath” module. 2) The inverted image will then be converted into a grayscale image that combines the red and green color channels, using the “ColorToGray” module. In almost all image analysis projects, one of the first steps will involve converting colored images into grayscale images because this step simplifies the algorithms by removing unnecessary information and reducing computational demand [18]. Additionally, all subsequent object identification modules in CellProfiler will only accept grayscale images. 3) To mask the EBD+ cells, we used the “ImageMath” module again to square the values of all the pixels in the grayscale image (Figure 1D). This operation darkens the gray pixels (reduces their pixel intensities), while keeping the intensities of the lighter colored pixels close to 1. In doing so, the dark gray pixels will be ignored in the subsequent object detection step. 4) The “IdentifyPrimaryObjects” module was then used to identify all the muscle cells which do not present any EBD staining (we refer to these as the Not_EBD_Cells) (Figure 1E). Setting the minimum and maximum diameter of muscle fibers filtered out objects that didn’t meet the diameter criteria. To determine the appropriate thresholding algorithm (which classifies pixels into foreground and background) analyzing the pixel intensity distribution with a histogram in CellProfiler can be helpful. Since our immunofluorescent images showed one peak at a low pixel intensity, we selected a robust background as our thresholding method. The robust background algorithm works by first removing the brightest (5%) and dimmest (5%) pixel intensities, as well as the foreground pixels leaving behind all the pixels that represent the background, since the background distribution approximates a Gaussian (CellProfiler Documentation 4.2.1, n.d.). The mean and standard deviation of the background pixel intensities are then calculated. From here, the threshold is calculated as the mean + N standard deviations. For our pipeline, we chose to set the threshold as the mean + 2 standard deviations, which is the standard value/ default value set by CellProfiler, in order to make the threshold more lenient in identifying the foreground pixels. However, this value can be adjusted to meet the needs of the image dataset such that when increasing the number of standard deviations will make the threshold more stringent compared to a low number of standard deviations. 5) Next, the pipeline retrieved the original immunofluorescent image and converted it into separate grayscale images representing each of the color channels using the “ColorToGray” module (Figure 1F). 6) The green channel grayscale image was then selected and inverted using the “ImageMath” module (Figure 1F). Inverting the green channel image makes the muscle cells (objects) appear light and the laminin appear dark. 7) Then, the “IdentifyPrimaryObjects” module was used again to identify all the muscle cells (including the EBD+ cells) in the image (Figure 1F). We again used the robust background algorithm to automatically calculate the threshold for each image. 8) The “CalculateMath” module was used to subtract the number of Not_EBD_Cells from the total number of muscle cells to determine the number of EBD+ cells in the image. 9) Lastly, the data collected was exported to comma-delimited files (.csv) using the “ExportToSpreadsheet” module.

**Figure 2:**
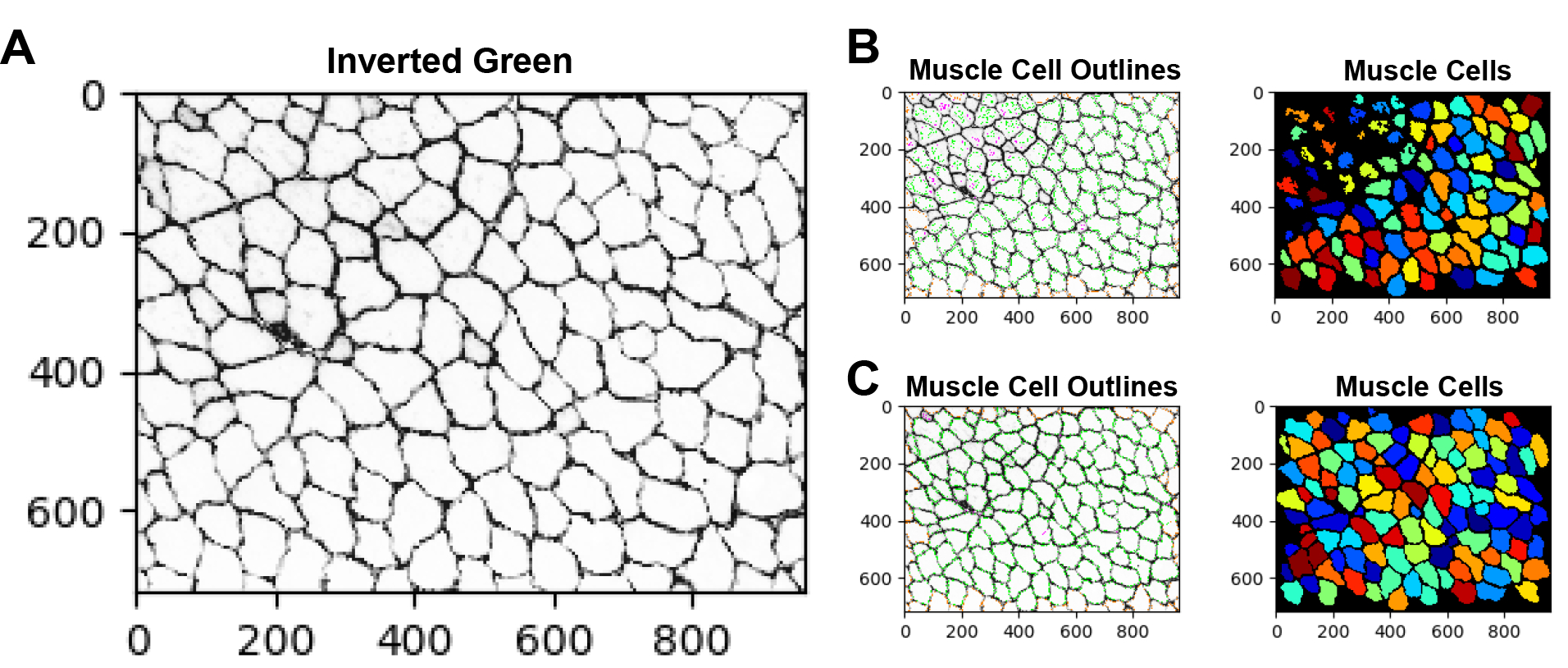
Examples of correct and incorrect image thresholding. Improper thresholding in the “IdentifyPrimaryObjects” module can impair object detection. **A)** The inverted green channel (grayscale) image used for muscle cell identification. **B)** Poor muscle cell identification. **C)** Improved muscle cell identification was achieved by adjusting the upper and lower bounds of the threshold.

There are several approaches that can be implemented to improve the accuracy of the EBD quantification. For example, adjusting the smoothing filter within the “IdentifyPrimaryObjects” module can be used to optimize object segmentation. This practice can be particularly useful for segmenting the dystrophic muscle fibers which are typically irregular in size and shape. If objects are over-segmented (when one object is incorrectly identified as multiple objects), it is recommended to increase the size of the smoothing filter. However, if the objects are under-segmented (when two or more objects are identified as a single object), then reducing the smoothing filter may be necessary. Additionally, when performing the same analysis on a new dataset it may be necessary to update some of the settings in the “IdentifyPrimaryObjects” modules. For example, Figure 2A and 2B shows a WT gastrocnemius muscle cross-sectional image with poor muscle cell detection. Here, the “IdentifyPrimaryObjects” module did not identify some of the muscle cells in the top corner of the image. By adjusting the upper and lower bounds of the threshold used for object detection we were able to improve muscle cell recognition (Figure 2C). Object identification is often the most challenging step in image analysis but is one of the most critical because its accuracy will determine the accuracy of subsequent measurements [19]. The test mode in CellProfiler can be used as quality control to ensure that all parameters are set up appropriately for the image dataset.

### 3.2 Quantification of Muscle Fiber Regeneration Using Embryonic Myosin Heavy Chain (eMyHC) Staining

**Figure 3:**
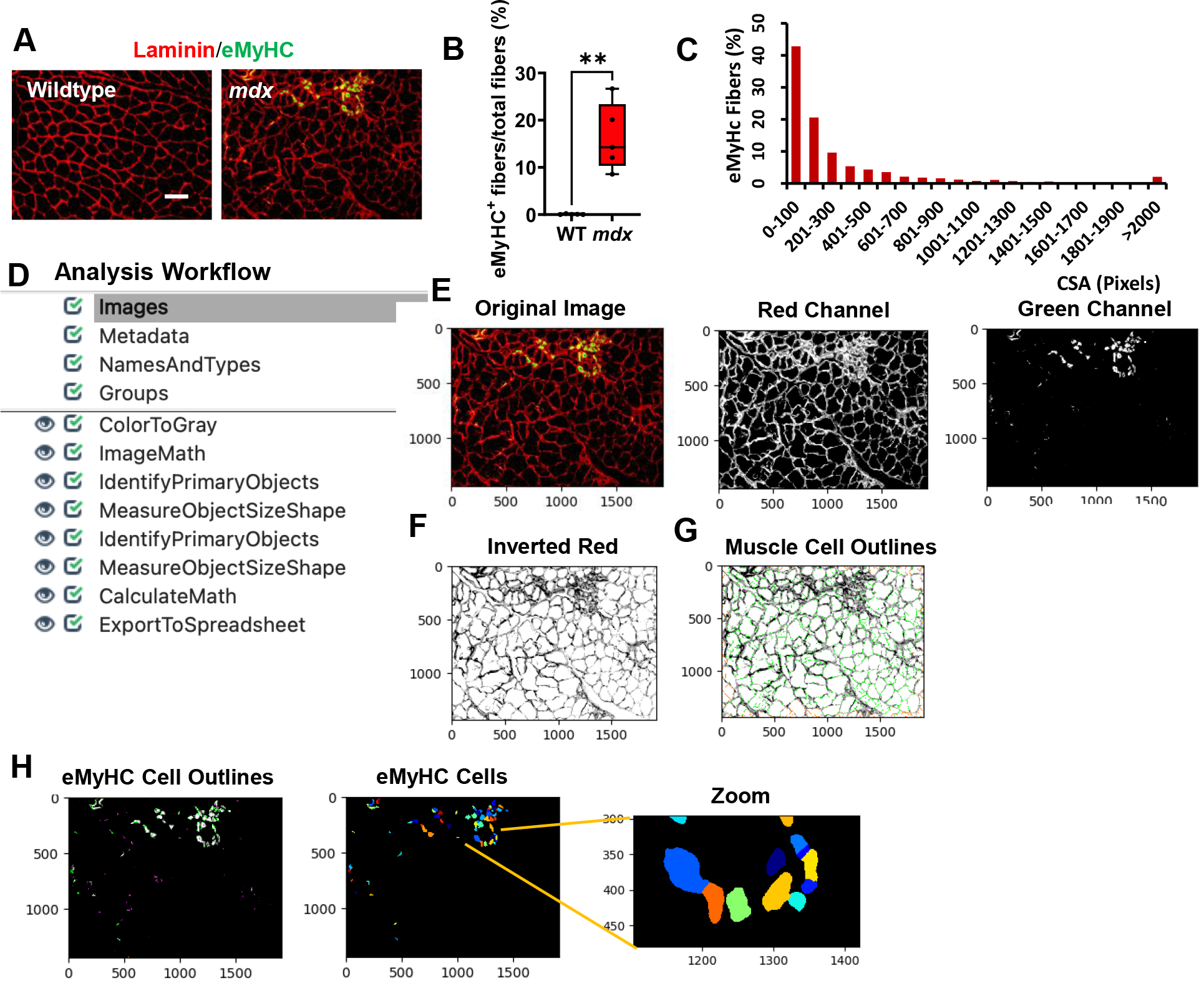
Processing and quantification of eMyHC+ myofibers by CellProfiler. **A)** Representative images of gastrocnemius muscle cross-sections from 4–6-month-old B10 and *mdx* mice immunolabeled with laminin α1 (red), and eMyHC (green). Scale bar = 100 μm. **B)** Boxplot displaying quantification of the sample eMyHC-positive muscle fiber expressed as the percentage of the total number of muscle fibers in WT and mdx mice (n = 5 mice/group). **p<0.01. **C)** Histogram showing the size distribution (in pixels) of the eMyHC+ fibers obtained from the sample dataset. **D)** Overview of the image analysis workflow. **E)** A sample *mdx* image was fed into the pipeline and converted into separate grayscale images representing each of the color channels. Here, only the red and green channels were used for subsequent processing. **F)** The red channel grayscale image was inverted and used for the **G)** identification of the muscle cells. **H)** Outlines of the identified eMyHC+ cells.

Skeletal muscle regeneration is sustained to counteract muscle degeneration. To detect muscle regeneration in our mouse models, we co-stained gastrocnemius muscle cross-sections from WT and *mdx* mice with laminin, and eMyHC (Figure 3A). All images were saved as TIFF files. After quantifying our sample dataset, we found that approximately 14% of the myofibers in the *mdx* images were eMyHC+, while the WT images showed no eMyHC+ myofibers (Figure 3B, *p value* 0.0079). Additionally, we found that the majority of these eMyHC+ myofibers were relatively small in size (Figure 3C). An overview of the muscle regeneration quantification pipeline is shown in Figure 3D. The eMyHC pipeline can be downloaded from the Supplementary Materials section (Supplementary File S2). A description of the eMyHC quantification workflow is provided below:

1) Once an image has been loaded into the pipeline, the image will first be converted into separate grayscale images representing each of the color channels using the “ColorToGray” module (Figure 3E). 2) Since the objects to be detected must appear lighter than the background, the “ImageMath” module was used to invert the red channel grayscale image (Figure 3F). 3) With the muscle cells appearing light and the laminin appearing dark, the “IdentifyPrimaryObjects” module was then used to identify the muscle cells (objects) (Figure 3G). We again used the robust background algorithm to threshold the images since these images mostly consisted of background. The size of the smoothing filter for “declumping” had to be optimized to the dataset to ensure proper segmentation of the muscle cells. 4) Next, the “MeasureObjectSizeShape” module was used to obtain area measurements for the identified muscle cells. 5) To detect the eMyHC+ cells, we used the “IdentifyPrimaryObjects” module again, but this time used the green channel grayscale image as input (Figure 3H). The green channel grayscale image did not need to be inverted prior to object detection, because the objects (eMyHC+ cells) already appeared light in a dark background. 6) The sizes of the eMyHC+ fibers were determined using the “MeasureObjectSizeShape” module. 7) Lastly, all the data collected were exported into separate spreadsheets using the “ExportToSpreadsheet” module.

### 3.3 Quantification of Muscle CD68 Deposition

**Figure 4:**
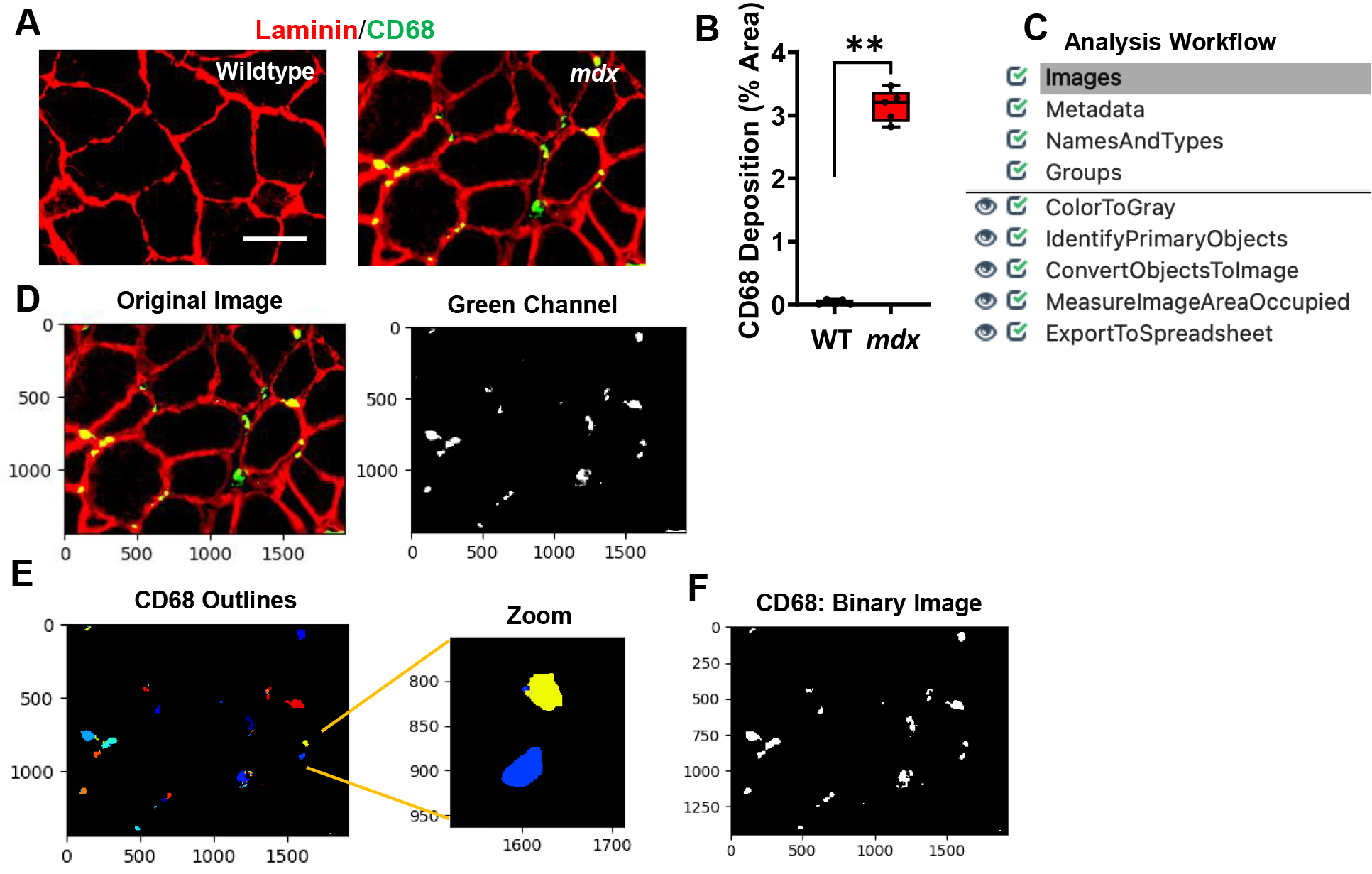
Processing and quantification of inflammation via detection of CD68 deposition by CellProfiler. **A)** Representative images of gastrocnemius muscle cross-sections from 4–6-month-old B10 and *mdx* mice immunolabeled with laminin α1 (red), and CD68 (green). Scale bar = 100μm. **B)** Boxplot illustrating the quantification of the sample CD68 dataset images showing the percent area of CD68 deposition in each gastrocnemius muscle of WT and mdx mice (n = 5 mice/group). **p< 0.01. **C)** Overview of the image analysis workflow. **D)** A sample *mdx* image was fed into the CD68 quantification pipeline which first converts the color image into separate grayscale images representing each color channel. The green channel grayscale image was used for further processing. **E)** Outlines of the identified CD68. **F)** The identified objects (CD68) were then converted into a binary black and white image for easy quantification of CD68 deposition (% area) within the muscle cross-section.

Inflammation is a pathological hallmark of muscular dystrophies, and dysregulated macrophages produce cytokines to promote remodelling within the dystrophic muscle [20, 21]. The infiltration of CD68+ M1 macrophages play a critical role in mediating the inflammatory response [14]. Gastrocnemius muscles from *mdx* and WT mice were stained with CD68, and laminin (Figure 4A). We found widespread CD68+ M1 macrophages in the *mdx* muscle cross-sections that formed clusters or aggregates. Since macrophage forms aggregates in some areas and it is nearly impossible to determine the number of CD68+ M1 macrophages within these aggregates, we quantified CD68 deposition by measuring the area of coverage in the muscle cross-sections. After quantifying the sample dataset, we found that approximately 3.2% of the area in *mdx* images consisted of CD68 deposition, whereas the WT images had hardly detectable CD68+ macrophages (Figure 4B, *p value* 0.0079). An overview of the CD68 quantification pipeline is shown in Figure 4C and can be downloaded from the Supplementary Materials section (Supplementary File S3). All images were saved as TIFF files and quantified using the following workflow:

1) The input immunofluorescent images were first converted into separate grayscale images representing each of the color channels using the “ColorToGray” module (Figure 4D). 2) The green channel grayscale image (representing the CD68) was used as input for the “IdentifyPrimaryObjects” module to identify the CD68 (Figure 4E). Here, we applied a global thresholding strategy and used the minimum cross-entropy algorithm to automatically calculate the threshold for each input image. Minimum cross-entropy uses the distribution of pixel intensities that define foreground and background as estimates for the probability distributions that produce the intensities of foreground and background. The cross-entropy between foreground and background is then calculated for each possible threshold. The final chosen threshold is the lowest cross-entropy value. 3) Next, the identified objects (CD68) were converted into a binary image using the “ConvertObjectsToImage” module (Figure 4F). Here, the white pixels represent the CD68, while the black pixels represent the background. Since white pixels have an intensity value of 1 and black pixels have an intensity value of 0, the creation of this binary image allows for simple quantification of the area occupied by CD68. 4) Using the “MeasureImageAreaOccupied” module the area covered with CD68 was determined 5) and the data was exported into .csv files using the “ExportToSpreadsheet” module.

### 3.4 Quantification of Collagen Deposition

**Figure 5:**
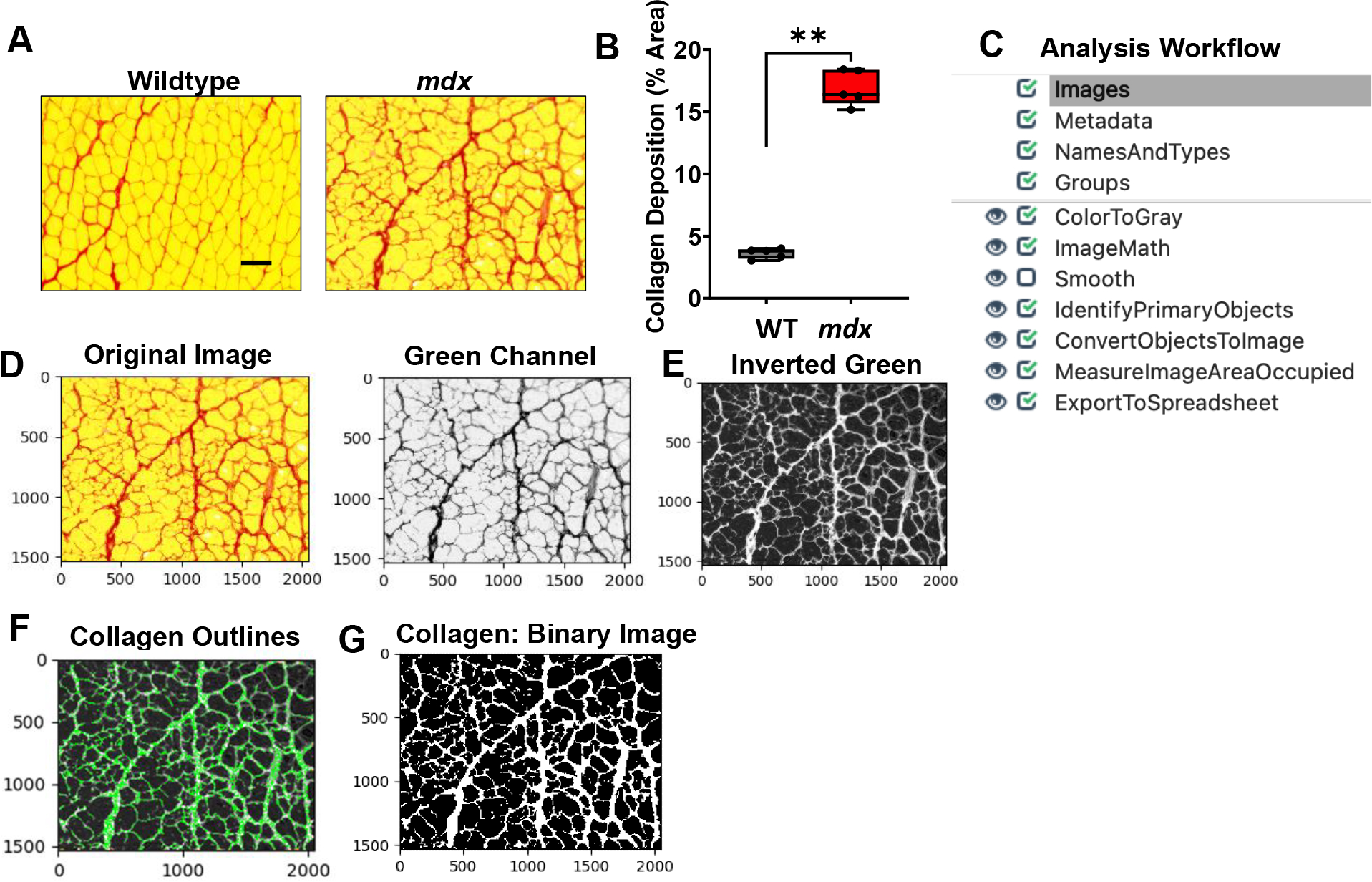
Processing and quantification of collagen deposition by CellProfiler. **A)** Representative images of gastrocnemius muscle cross-sections from 4–6-month-old B10 and *mdx* mice stained with Picrosirius red. Scale bar = 100μm. **B)** Boxplot illustrating quantification of percent area of collagen deposition in each muscle section (n = 5 mice/group). **p<0.01. **C)** Overview of image analysis workflow. **D)** A sample *mdx* image was fed into the collagen quantification pipeline, which first converts the color image into separate grayscale images representing each of the color channels. The green channel grayscale image was selected for subsequent processing because the collagen had a prominent appearance in this channel. **E)** The green channel image was inverted and used for **F)** identification of the collagen. **G)** The identified collagen was then converted into a binary black and white image for simple quantification of collagen deposition (% area) within the muscle cross-section.

The heightened inflammatory response observed in the dystrophic muscle eventually leads to excessive collagen deposition which causes muscle stiffness and functional deficit [12]. Collagen deposition was detected through Picrosirius red staining of gastrocnemius muscle cross-sections from WT and *mdx* mice (Figure 5A). We found a significant increase in collagen deposition in the *mdx* cross-sections compared to the WT muscle cross-sections (Figure 5A & 5B, *p value* 0.0079). An overview of the collagen quantification pipeline is shown in Figure 5C and can be downloaded from the Supplementary Materials section (Supplementary File S4). All images were saved as TIFF files, and quantified as described below:

1) Images loaded into the collagen quantification pipeline were first converted into separate grayscale images using the “ColorToGray” module (Figure 5D). The green channel grayscale image was selected for further processing because this channel showed the collagen most clearly. 2) The “ImageMath” module was then used to invert the green channel image to ensure that the desired foreground (collagen) appeared light, while the background (muscle cells) appeared dark (Figure 5E). 3) Next, the “IdentifyPrimaryObjects” module was used to identify the collagen (objects) surrounding the muscle cells (Figure 5F). For our dataset, we chose to set the values of the typical minimum and maximum diameters of the objects to represent the size of myofibers. Additionally, we used the minimum cross-entropy thresholding algorithm to automatically calculate the threshold used for each image. 4) The identified objects (collagen) were then converted into a binary image using the “Convert Objects to Image” module (Figure 5G). As previously mentioned, the creation of this binary image simplifies the subsequent image area measurement. 5) The “MeasureImageAreaOccupied” module was then used to measure the collagen pixel area. 6) Lastly, the measurements were exported into .csv files and imported into Excel for further analysis. To determine the percentage of muscle area occupied by collagen, the collagen area was divided by the total area represented in each image.

## 4. Discussion

Detection, characterization, and quantification of muscle pathology provide valuable information which can be used to monitor the progression of muscle disease or even evaluate the effectiveness of treatment intervention. Although this phenotypic data is an essential component of muscle research, many individuals currently rely on manual or semi-manual methods to obtain this data which is not only labor-intensive but can potentially introduce inter-individual bias. A previous study by Lau et al., 2018 [22] showed that CellProfiler could be utilized to quantify the size distribution and central nucleation of muscle cells. Here, we expanded upon their approach to obtain a more comprehensive analysis of skeletal muscle health, focused on muscle membrane damage, the size, and abundance of regenerating muscle fibers, muscle inflammation, and collagen deposition.

Extensive muscle membrane damage is a hallmark of muscular dystrophy that can be accessed via EBD staining [7]. We established an EBD pipeline to quantify the number of muscle fibers that exhibit membrane instability. When we designed the EBD pipeline, we originally planned to use the red channel to identify the EBD+ cells. However, since the shapes of damaged muscle cells are poorly defined, we found it particularly difficult to accurately segment these objects. Due to this challenge, we opted to mask the EBD+ cells by reducing their pixel intensities and instead decided to identify all the Not_EBD_Cells and subtract this quantity from the total number of identified cells to give the number of EBD+ cells. Once damage has occurred in skeletal muscle, muscle cells have the ability to regenerate. To quantify the size and abundance of the regenerating muscle fibers, we followed a similar approach for the eMyHC+ cells but did not need to mask the eMyHC+ cells because these cells had a well-defined shape. The eMyHC+ cells were directly detected and measured using the green channel from the immunofluorescent images.

Compared to the rare and dispersed distribution of CD68+ M1 macrophages in healthy muscle, the *mdx* muscle exhibits large aggregates of these macrophages which are often found in necrotic regions. Since the individual CD68+ M1 macrophages that compose these clusters are poorly defined, we found it difficult to accurately determine the number of these pro-inflammatory macrophages in each cluster. Due to this difficulty, we quantified inflammation by measuring the area of the muscle cross-section occupied by the CD68+ M1 macrophages. To evaluate muscle fibrosis, we used a similar approach in our collagen pipeline to quantify the area of collagen coverage in the muscle cross-sections.

Before getting started with CellProfiler, there are a few important considerations to keep in mind. Because of tissue folding or tearing, poor laminin staining around the muscle cell boarders, some extra regions appeared incorrectly assigned to the morphology of muscle fiber, and the presence of background staining can all potentially induce quantification errors. To minimize the rate of these errors, high quality images are critical for accurate quantification. In addition, carefully adjusting the parameters for thresholding can greatly reduce the rate of errors. In addition, we recommend to add secondary antibody-only controls to optimize the imaging parameters.

To validate the muscle cell identification accuracy in our EBD and eMyHC pipelines, we compared CellProfiler’s automated quantification with manual quantification. After using both methods to quantify our sample datasets, we found that the automated quantification was very similar as manual quantification (data not shown). To test the reproducibility of Cell Profiler, analysis was also done by different users with different levels of experience in these types of analyses. Different users obtained very similar results.

It should be mentioned that automated quantification can avoid inter-individual bias introduced by manual quantification of microscopy images, allow for higher analysis throughput, and provide reproducibility and rigor of histological studies. Quantification using CellProfiler will improve the reproducibility of microscopy image quantification. Furthermore, CellProfiler can not only produce reliable quantification but does so in a time efficient manner. While it took hours and days to manually quantify our sample EBD and eMyHC datasets, CellProfiler was able to perform the same quantification within one minute. Several software including Fiji and ImageJ have been shown to be able to perform quantification analysis. However, CellProfiler enables large-scale modular image processing without the need for any prerequisite knowledge of programming languages. In addition, it is a free open-source software that is available for both Windows and macOS.

Although the present study focused on the quantification of gastrocnemius muscle cross-sections, the workflows we generated can be applied to much wider applications. In fact, the same quantifications could be conducted on a variety of other muscles and tissues. For example, inflammation and fibrosis are common features of many other chronic diseases, affecting a variety of tissues. The CD68 and collagen pipelines shown here are not limited to muscle tissue and could be used to quantify inflammation and fibrosis in essentially any kind of tissue section. In summary, the image analysis pipelines presented here not only perform histopathological quantification in a time effective manner but can be used to quantify even the subtle features of microscopy images that aren’t easily detected by the human eye [19].

## 5. Conclusions

Images can provide a wealth of phenotypic data but extracting this data via manual quantification is not only subjective, but also time-consuming [23]. The present paper describes four image analysis pipelines utilized to evaluate skeletal muscle cross-sections for membrane instability, muscle regeneration, inflammation, and collagen deposition. Computer-based image analysis offers many advantages over manual analysis including reproducibility, simultaneous image processing, ability to measure and extract multiple features in a single run, as well as adaptability. Our tools should aid in the analysis of muscle disease progression and evaluation of therapeutic intervention by facilitating faster more accurate data acquisition.

## Supplementary Material

Supplementary Supplementary File S1: Pipeline for EBD quantification
Supplementary File S2: Pipeline for eMyHC quantification
Supplementary File S3: Pipeline for CD68 quantification
Supplementary File S4: Pipeline for collagen quantification

## Data Availability

The pipelines used to generate all data for this study are included in the supplementary material file.

## List of abbreviations

EBD: Evans blue dye
WT: wild-type
eMyHC: embryonic myosin heavy chain
DMD: Duchenne muscular dystrophy
OCT: optimal cutting temperature
